# Interplay of Spatial Structure and Interactions in Microbial Communities

**DOI:** 10.1111/1462-2920.70262

**Published:** 2026-03-02

**Authors:** Vaishnavi Warrier, Yilin Chen, Ethan Rappaport, Shin Haruta, Hyun Youk, Babak Momeni

**Affiliations:** ^1^ Biology Department Boston College Chestnut Hill Massachusetts USA; ^2^ Department of Biological Sciences Tokyo Metropolitan University Tokyo Japan; ^3^ Center for Biophysics and Quantitative Biology University of Illinois Urbana‐Champaign Urbana Illinois USA; ^4^ Department of Physics University of Illinois Urbana Champaign Urbana Illinois USA

## Abstract

Given that most microbes experience spatially structured environments, examining how such environments affect microbial growth and functions is paramount. Previous studies have shown that a spatially structured environment can impact microbial growth and interactions, and that microbial growth can create or magnify spatial structure. Here, we review some of these instances of past studies to develop a consistent framework that highlights the interplay between microbial interactions, spatial structure of the environment and spatial organisation of microbes. We re‐examine the level, degree and scale of spatial structure with regard to the phenomena and biological processes of interest. We then discuss how mathematical models can reveal the contribution of the spatial structure to community assembly and coexistence. Lastly, we offer an outlook on important steps for the progress of this field.

## Graphical abstract

A description of the interplay between microbial growth and interactions, the spatial structure of the environment and the spatial organisation of microbial populations can improve our understanding of the ecology of microbial communities. We review previous studies that highlight different aspects of this interplay.

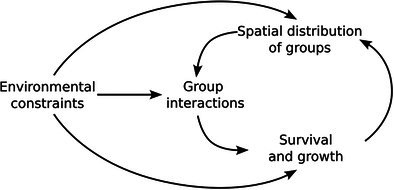

## 1 Introduction

### 1.1 What Is Spatial Structure?

In the context of microbial ecology and evolution, a spatially structured environment is one in which individual microbes experience different local conditions (Durrett and Levin 1994). This environment can include abiotic factors, such as temperature (Dal Bello and Abreu 2024), oxygen level (Fenchel and Finlay 2008; Borer et al. 2018) or physical barriers, or biotic factors, such as the physical presence of other microbes (Kolenbrander et al. 2010) or a gradient of their metabolic byproducts (Michielsen et al. 2024; Duxbury et al. 2025). A spatially structured environment is defined in contrast with a uniform (also called homogeneous or well‐mixed) environment in which all individual microbes experience exactly (or almost exactly) the same conditions in their environment (Durrett and Levin 1994). As one would expect, most microbes live in spatially structured environments. Individual microbes may experience different environments for several reasons. The environment may not be uniform (Rainey and Travisano 1998), and individuals may experience different levels of temperature, pH, nutrients or oxygen availability, for example. Within a biofilm, each cell may experience different local conditions, simply due to having different numbers and types of neighbouring cells around it (Wimpenny et al. 2000). Some microbes may be spatially associated with other microbes, for example, because of physical aggregation (Kolenbrander et al. 2006). Even when there may not be any exchange of molecules between two microbes, the competition for locally available nutrients means that one microbe affects other microbes in its vicinity. In this sense, living in a spatially structured environment is the norm rather than the exception.

### 1.2 The Level and Degree of Spatial Structure

The impact of spatial structure can be examined at different levels (Borisy and Valm 2021), from individual‐level, that is, the influence between individual cells, to group‐level, that is, the net effect between groups or populations of cells. Each group designates a specific ‘type’ of cell which, depending on the context of interest, may refer to a group of individuals in a community, a group with a certain genetic or phylogenetic similarity (such as a phylum, a genus, a species, or a strain), or even a group of individuals with a certain phenotypic trait (Vlamakis et al. 2008). For the majority of our following discussions, we choose to focus on the group‐level scale. With this choice, the impact of a spatial environment is still represented but is simplified into net effects on groups, allowing direct comparison with well‐mixed counterparts. For each group, we define its spatial distribution as how the individuals within that group are spread across space. The spatial distribution of each group is often the product of many different factors, from environmental influences and extracellular matrix production to interpopulation interaction and cell motility (Booth and Rice 2020). Motility, in particular, can have a profound impact on the spatial distribution of groups, especially when considering processes such as chemotaxis, collective response or quorum sensing (Mohanty and Firtel 1999; Ben‐Jacob et al. 2000; Parsek and Tolker‐Nielsen 2008).

The presence or absence of spatial structure conceptually is defined based on whether individuals within a group experience the same environment (Durrett and Levin 1994). Considering that the mere presence of other individuals changes the environment experienced by each individual (e.g., in a colony), it is clear that the absence of spatial structure is only an abstract theoretical possibility. However, the degree of spatial structure matters. For example, whether individuals from one group are clustered with their own type or are distributed among a partner group can influence access to resources and inter‐group interactions.

Several metrics have been defined in ecological studies to quantify the degree of spatial organisation of organisms, resources and environmental factors. These metrics can be broadly divided into three groups: (1) Spatial autocorrelation metrics (Getis 2010), such as Moran's I or Geary's C, which quantify the degree of similarity or dissimilarity at nearby spatial locations to represent the degree of clustering or dispersion. (2) Point pattern analysis metrics (Ben‐Said 2021), such as nearest neighbour distance analysis or Ripley's K function, to assess whether the distribution of individuals in the domain of interest is random or structured. (3) Landscape ecology metrics (Francis et al. 2022), such as metrics for patch size, shape, distribution and connectivity, which measure how patches are spread across a landscape. In practise, a modified version of such metrics is often used, tailored to the biological phenomenon of interest. For example, Dang et al. (2020) and Maire and Youk (2015) introduced a spatial correlation function weighted by the communication strength between every pair of cells to represent a spatial index *I*. This single number captured the degree of order in the spatial structure, as related to the spatial self‐organisation of cells communicating via one or more diffusible ligands.

### 1.3 The Scale of Spatial Structure

Related to the idea of the degree of spatial structure is the scale of biological processes in a community. Temporal and spatial scales are often of particular interest—the relevant time over which the biological processes of interest occur and the relevant spatial reach of these processes. The relevant scales can be substantially impacted by the underlying physical processes that are in effect, such as the spread of nutrients in an environment by diffusion versus by flux or transport. Depending on the underlying processes, the temporal and spatial scales can also be linked. For example, two processes commonly governing metabolite‐mediated interactions are diffusion and transport. If an interaction is driven by a diffusion process, the temporal scale and spatial scale are related as $L_{\text{diff}}\approx 2\sqrt{Dt_{\text{diff}}}$ ($L_{\text{diff}}$: length scale of diffusion; D: diffusion coefficient; and $t_{\text{diff}}$: time scale of diffusion). In contrast, with flux or transport processes, $L_{\text{flux}}\approx vt_{\text{flux}}$ ($L_{\text{flux}}$: length scale of flux/transport; v: transport velocity in the environment; $t_{\text{flux}}$: time scale of flux/transport). It is important to note that such relationships can have profound consequences. For instance, if a metabolite spreads in space by diffusion, it is unlikely for it to reach long distances within relevant time scales. Assuming a relatively large diffusion coefficient of $D=300\mu m^{2}/s$, diffusion takes a metabolite a distance of 100 μm, 1 mm and 10 mm approximately in 15 s, 25 min and 42 h, respectively. In contrast, the time required for transport linearly scales with distance. Ultimately, the temporal and spatial scales relevant to the biological processes/variables of interest will determine whether the scale of a spatial environment is impactful. For example, a 100‐μm scale habitat heterogeneity would make the environment spatial when the relevant biological processes have a spatial scale of 10 μm, but not when they have a spatial scale of 50 mm.

Previous reports have also examined the consequences of spatial scale. For instance, Hart et al. (2017) examined whether a certain spatial scale is required to maintain species diversity. They introduced the coexistence‐area relationship as a practical measure to represent how coexistence can be driven by the spatial scale of the environment. Examining motility, Song et al. (2009) have shown that when interactions had the same spatial scale as the spatial distributions, increased motility negatively impacted diversity in an engineered model of interpopulation exploitation. In a system of cooperators and cheaters, Luo et al. (2024) showed that a structured environment during range expansion might suppress cooperation by allowing cheaters to more effectively exploit the cooperators.

### 1.4 Functional Consequences of Spatial Structure

From a practical point of view, the motivation behind examining spatial structure is to understand its functional consequences. Some impacts of spatial structure on microbial communities and their functions are intuitive. It is easy to imagine how restricting the access of interaction partners to each other affects the outcomes; notably, as Gause showed, prey–predator coexistence may depend on the ability of prey to avoid the predator in a spatial refuge (Gause 1934). Later, it was shown that a simple spatial structure of two connected patches might be enough to allow the prey to stably persist in a predator–prey system (Jansen 1995). Population/group distributions can also be functionally consequential. For example, wastewater microbial granules have layers of different microbial populations/groups and successively break down complex compounds; such granules will not work as effectively if the order of species is reversed (Satoh et al. 2007). Spatial structure can also change the intensity of competition. This has been shown in plant systems, when slow growers could persist in a spatial environment in direct competition with fast growers, if the slow growers were better at colonising new territory compared to fast growers (Tilman 1994). In a bacterial system, Gude et al. (2020) showed using two wild isolates of *E. coli* that, in a spatial environment, a trade‐off between motility and competitive advantage could allow bacterial populations to coexist. In their study, one strain had a growth advantage over the other strain and dominated well‐mixed cultures, regardless of the initial ratio of the two strains. When grown in low‐density agar gels, however, the more motile, slow‐grower strain could outcompete the less motile, fast‐grower strain when the slow‐grower strain was in low relative frequency, as it could expand and occupy more territory (Gude et al. 2020). Several additional examples that highlight the impact of spatial structure are reviewed nicely in (Tolker‐Nielsen and Molin 2000).

Spatial structure has been found to influence species interactions in several empirical studies. We highlight a few past examples, noting that these examples are chosen not to comprehensively represent the prior work but to bring up some of the underlying mechanisms and concepts. Kim et al. (2008) used microfluidic chambers connected via channels to assess how spatial separation between populations can affect coexistence. They used three populations that could benefit each other: a nitrogen fixer (providing amino acids to others), a cellulose degrader (providing a usable carbon source to others) and an antibiotic‐degrader (removing a harmful antibiotic). They observed that when the spacing was too short (mixed in the same well), competition dominated and disrupted coexistence, and when the spacing was too long (1.8 mm), facilitative interactions became too weak to sustain coexistence; coexistence was achieved at intermediate spatial spacing between these extremes (0.6 and 1.2 mm) (Kim et al. 2008). Harcombe et al. (2014) examined how the spatial arrangement of different populations affected their interactions. They used a methionine‐requiring strain of *E. coli* that cooperated with a methionine‐excreting strain of *Salmonella* that itself benefited from acetate produced by *E. coli* . They observed that metabolic interactions between these two partner populations may be intercepted by a methionine non‐producer strain of *Salmonella* that was spatially situated between the two partners—they referred to this phenomenon as the metabolic ‘eclipse’ effect (Harcombe et al. 2014). In our own prior work, we have shown that in a system of cooperators and cheaters, spatial self‐organisation driven by species interactions can favour cooperation and disfavour cheating (Momeni, Waite, and Shou 2013). Datta et al. (2013) explored how spatial structure at the expanding range of a community reshaped the distance between different phenotypes and thereby influenced their competition and the trajectory of evolution. In these studies, the role of spatial distance between interacting groups in modulating their interactions is noteworthy.

**FIGURE 1:**
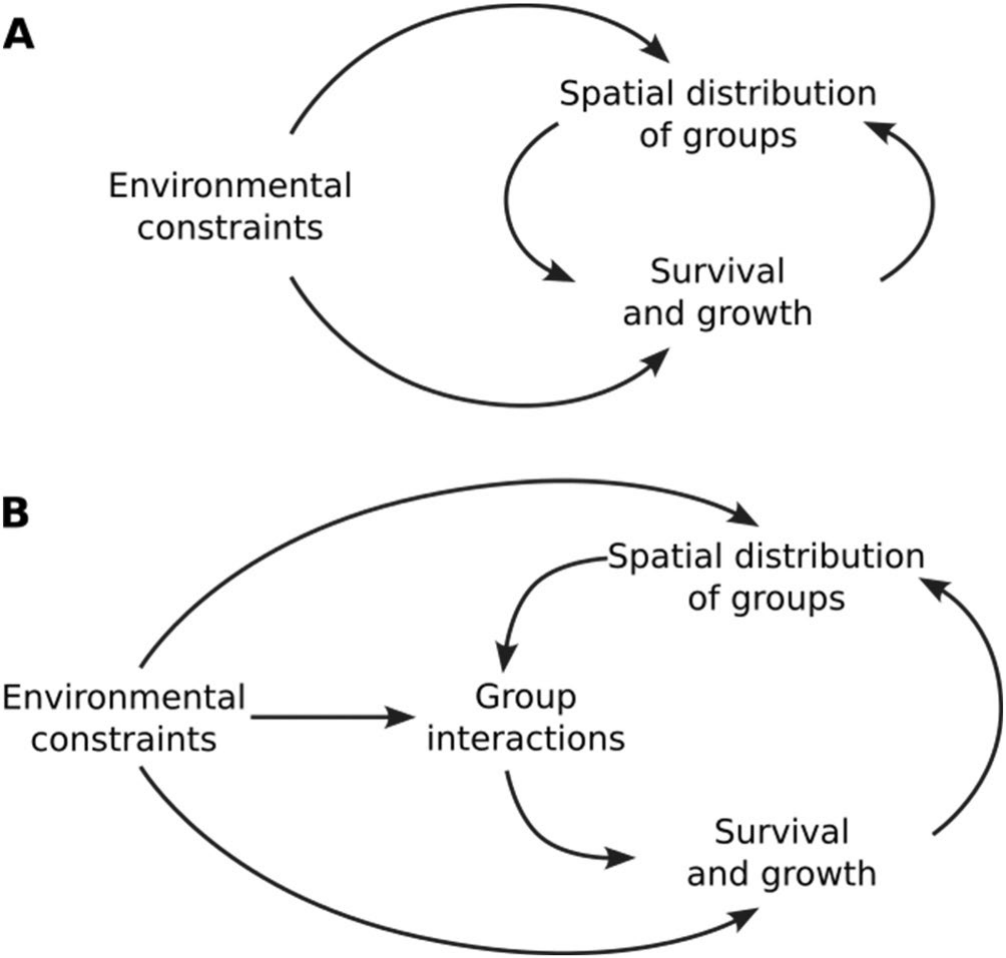
The interplay between biotic and abiotic factors drives the spatial distribution of groups within a community and affects community properties. (A) From a conceptual perspective, each group's survival and growth and the spatial distribution of groups mutually impact each other, with environmental constraints influencing both. (B) Inter‐group interactions—how groups within the community, on average, interact with each other depending on their spatial distributions—can be seen as an intermediate between spatial distribution and group survival and growth.

In the rest of this paper, we take the simplified view of examining the role of spatial structure in creating or modifying the feedback (Henderson et al. 2025) between the environmental constraints, the survival and growth of groups in those environments, and the resulting spatial distributions of the groups (Figure 1A). Taking one additional step towards a more mechanistic representation, we examine inter‐group interactions in the spatial context and how they might act as an intermediate in the interplay between the environment, growth and patterns (Figure 1B). The interactions, for example, by competition for resources, cross‐feeding, or release of inhibitory compounds, take place at a cellular level, but the ‘inter‐group interactions’ terminology refers to the net effect of interactions on a group across the community and depends on the spatial distribution of the interacting groups.

## 2 The Drivers of Spatial Structure in Microbial Communities

Spatial structure can be generated by abiotic or biotic processes. Abiotic structure might be the result of existing heterogeneity in the environment, which leads to individuals experiencing different environments, for example, across a gradient of oxygen (Ludemann et al. 2000), nutrients (Codeço and Grover 2001), or light (Martinez et al. 2019). In contrast, biotic spatial structure can be caused either by the physical presence of other organisms in the environment, by the heterogeneity in the environment caused by the activity of other groups, or even by group heterogeneity arising from noisy gene expression within an isogenic group. Competition for space in territorial groups (Weiner et al. 2019)—when cells occupy spatial locations and exclude others from those locations—can be considered an example of spatial structure generated by physical constraints, and the heterogeneous distribution of a beneficial mediator in commensalisms between two species (Momeni, Brileya, et al. 2013; Martinez‐Rabert et al. 2022) would be an example of spatial structure generated by the activities of the groups. These drivers of spatial structure rarely work in isolation. The spatial structure of a microbial community is often the result of several factors, both biotic and abiotic, that dynamically change and modulate each other. This intricate interplay makes spatial distribution patterns complex and context‐dependent. A well‐known manifestation of this interplay is the emergence of Turing patterns from a combination of short‐range facilitation and long‐range inhibition interactions (Karig et al. 2018; Oliver Huidobro et al. 2022).

From a different perspective, how the structure is generated can be categorised into two different types: guided versus self‐organised (also called non‐autonomous versus autonomous (Lu et al. 2022)). Guided spatial structure refers to situations in which the spatial distributions of groups are driven by external factors. These external factors could be imposed by the physical and/or chemical constraints of the environment, including abiotic environmental heterogeneity, or by signals and/or cues generated by groups within the community. As an example, an external oxygen gradient could restrict the presence of some anaerobic oral bacteria to regions below the gum line in the mouth (Fine and Schreiner 2023). Self‐organised spatial structure can be either internally coordinated or interaction‐driven. Internally coordinated spatial structure refers to situations in which the spatial distribution of a group is driven by an evolutionary adapted genetic programme. Conceptually, this is akin to coordinated patterns developing in multicellular organisms (O'Toole et al. 2000). Examples of internally coordinated self‐organised spatial patterns include the phenotypic differentiation in *B. subtilis* biofilms (Vlamakis et al. 2008; van Gestel et al. 2015), metabolic differentiation in *S. cerevisiae* colonies (Cáp et al. 2009), or cell differentiation in stalk‐like structures of *S. cerevisiae* (Scherz et al. 2001). Interaction‐driven self‐organised spatial structure refers to situations in which spatial patterns emerge from interactions among groups (Johnson and Boerlijst 2002). Examples of interaction‐driven self‐organised spatial patterns include the spatial intermixing of mutualists (Momeni, Brileya, et al. 2013), spatial isolation of cheaters (Momeni, Waite, and Shou 2013), spatial isolation of antibiotic‐sensitive members from antibiotic‐producers (Narisawa et al. 2008), or engineered patterns in synthetic populations (Basu et al. 2005). A defining feature of interaction‐driven self‐organised spatial structure is that it can be explained by intra‐ and inter‐group interactions among the constituents of the community, without necessarily involving evolutionarily selected programmes that drive the internally coordinated patterning. Patterning in interaction‐driven spatial self‐organisation is expected to be reproducible by other groups engaged in those interactions, whereas internally coordinated spatial organisation is expected to be unique to particular organisms or taxa. We still acknowledge that the distinction between different types of guided versus self‐organised patterns may not be clear in communities in which the interactions and molecular mechanisms are less well‐known.

In what follows, we will briefly survey some examples of driving forces that can generate spatial structure and influence interactions in microbial communities.

### 2.1 Abiotic Spatial Heterogeneity

**FIGURE 2:**
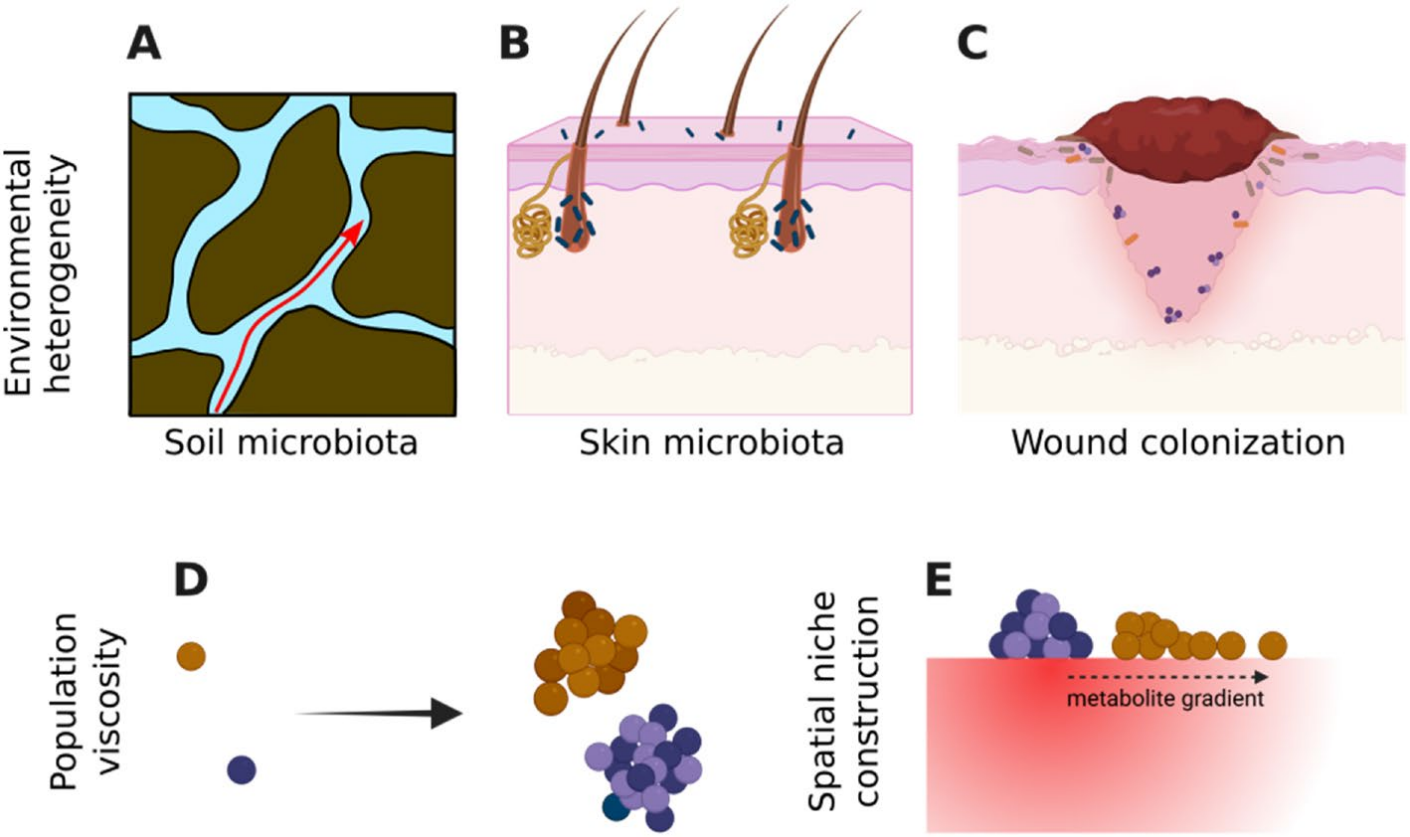
Abiotic and biotic spatial heterogeneity affects the spatial distribution of microbial groups. As representative examples, abiotic spatial heterogeneity is experienced by (A) soil microbes in water channels around soil particles, (B) commensal bacteria colonising skin glands and hair follicles and (C) pathogenic bacteria and archaea at the site of a skin wound. Biotic forces can also make the environment heterogeneous, for example, by (D) accumulation of progeny in the vicinity of parent cells due to slow dispersal or (E) creation of environmental gradients from species activities such as production of a diffusible metabolite.

The heterogeneous environment can take many forms. There are instances where the heterogeneity that microbes experience is intrinsic to the abiotic environment. For example, the porous soil structure generates a patchy environment (Raynaud and Nunan 2014) and affects the transport of water through the soil and subsequently impacts microbial interactions and functions (Figure 2A). On the surface of skin, glands and hair follicles offer heterogeneous shelter to bacterial populations (Figure 2B) (Grice and Segre 2011). Similarly, in bacterial colonisation of wounds, different zones in this heterogeneous environment allow different bacteria to persist (Figure 2C). Although in the last two examples the host is a living organism, from the perspective of microbes and for a limited timescale, it can be viewed as static, similar to an abiotic environment.

### 2.2 Biotic Spatial Heterogeneity via Population Viscosity

A major driver of biotic spatial structure is population viscosity—a phenomenon in which progenies remain physically close to their parents, being unable or slow to disperse away (Figure 2D). Often, such population viscosity leads to increased intra‐group interactions over inter‐group interactions (Taylor 1992). During range expansion of mixed groups, for example, this competition becomes an important factor in how different groups compete as they take over new territory (Martinez‐Rabert et al. 2022; Hallatschek et al. 2007; Korolev et al. 2012).

### 2.3 Biotic Spatial Heterogeneity via Niche Construction and Ecosystem Engineering

In many cases, the microbial groups themselves are a major contributor to the spatial structure of the environment. Conceptually, this can be viewed as niche construction, changing the environment experienced by those groups as well as other groups encountering that habitat (Figure 2E). For example, altruistic cell death and toxin release by a competitively disadvantaged strain can create a zone in which that strain can survive and prosper in a spatial environment (Chao and Levin 1981). A conceptually similar phenomenon is species creating spatial gradients of environmental resources and properties. For example, in a multispecies community that degrades cellulose, obligate aerobes in a static liquid culture can create a low‐oxygen zone for oxygen‐sensitive cellulolytic members to survive and contribute to the community function (Kato et al. 2005; Kato et al. 2004). Aerobic nitrifying bacteria and anaerobic ammonium‐oxidising bacteria work together and form microgranules with a layered structure (Chen et al. 2019). In oral microbiota, early‐colonisers create a spatial niche in which attachment of others to these early‐colonisers is a critical component of community development (Kolenbrander et al. 2010). Primary producer cyanobacteria can create anoxic zones in aggregates that support stable persistence of heterotrophs (Duxbury et al. 2025). Even more directly, the extracellular matrix generated by microbial species in biofilms can create the spatial structure within which other cells are distributed and organised (Nadell et al. 2016; Kolenbrander et al. 1999).

Overall, the spatial structure of a microbial community can have different sources. It can originate from external biotic or abiotic sources, from the activities of each group, from the presence or activities of other groups within the community, or a combination of these sources. Recognising which sources contribute to the spatial structure of a community can be important for better understanding how the spatial structure of the community establishes, persists or progresses.

## 3 How Interactions Are Affected by the Spatial Context

The spatial context can determine what groups within the community interact with each other, how often they can interact, and what outcomes emerge from those interactions. In a simplified view, access to the partner is the primary factor that directly modulates interactions. Spatial self‐organisation, driven by microbe‐environment and microbe‐microbe interactions, can also influence group distributions and subsequently affect interactions.

### 3.1 Access to a Partner

One of the primary ways that spatial structure affects community structure is through reshaping interaction opportunities among cells. Guided spatial structure can influence the chance of interactions between species. As an example, environmental compartments in Gause's study of prey–predator dynamics allow the prey to avoid the predator (Gause 1934). An intuitive example of species‐driven structure affecting access to partners happens with population viscosity: accumulation of progeny around dividing cells increases the likelihood of encounters and interactions within the same group and decreases the likelihood of interactions with other groups (Kanwal and Gardner 2022). The eclipse effect mentioned earlier and shown by Harcombe et al. (2014) underscores another facet of access, when a group intercepts the interaction mediators and blocks or weakens interactions between other groups.

A key determinant of access to the partner is the spatial distance between cells of each group from other cells of their own group or other groups. In their seminal work, Kim et al. (2008) used controlled experiments in connected microfluidic chambers to show that the strength of interactions could be modulated by changing the distance between groups. A clear manifestation of the role of distance is in the interspecies syntrophic hydrogen transfer in syntrophic methanogenic consortia (Ishii et al. 2006). The dependency of interactions on the spatial distance between groups can take different forms. Romdhane and colleagues conceptually categorise the dependency into three possibilities: independent, linearly decreasing and threshold‐based (Romdhane et al. 2024). For interactions that rely on metabolite exchange or influence, the linear dependence likely indicates that they can be associated with transport processes. In contrast, threshold‐based interactions are likely associated with diffusion processes, where long‐range interactions at relevant timescales are not expected (see Section 1.3).

### 3.2 Microbial Interactions Can Be Modulated by Spatial Self‐Organisation

**FIGURE 3:**
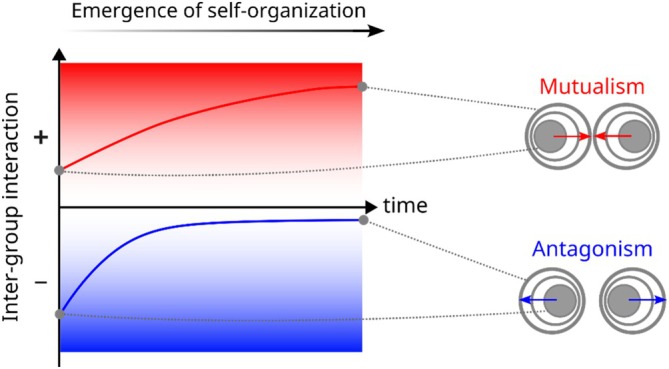
When spatial self‐organisation drives group distributions within a community, the effective strength of inter‐group interactions will change. In mutualism (top), since each group grows better in the vicinity of the partner group, over time, the groups become more spatially associated, and the effective mutualism interaction becomes stronger. In antagonism (bottom), each group grows poorly in the vicinity of the partner; thus, the groups become more segregated over time, and the effective antagonism interaction becomes weaker.

Self‐organised spatial structure—ordered spatial structure emerging from interactions within the community—can also change intra‐ and inter‐group interactions. These changes often follow certain trends based on the intrinsic feedback between group growth, spatial patterns and interaction‐driven dynamics. When driven by inter‐group interactions, spatial self‐organisation often favours facilitation and disfavours inhibition (Estrela and Brown 2013). This trend is intuitive, as the growth of each group in space will be favoured in regions that are closer to facilitatory partners and away from inhibitory ones (Figure 3). The emergence of patterns such as intermixing of mutualists, layering of commensals and segregation of competitors is the result of this trend (Momeni, Waite, and Shou 2013; Estrela and Brown 2018).

## 4 How Coexistence Is Affected by the Spatial Context

### 4.1 Niche Partitioning Enabled by Spatial Heterogeneity

Spatial context can be an important factor in permitting and maintaining coexistence. A primary concept through which coexistence is supported in a spatial structure is through niche partitioning (Amarasekare 2003). Niche partitioning is defined as the process by which different groups access different resources or niches within a community, often leading to reduced competition and more coexistence. As an example, spatial structure can support the coexistence of antagonistic competitors in a patchy environment, with each patch dominated by one or the other competitor (Hanski 1983). The weaker competitors can persist by occupying patches or microhabitats where they escape competition from other competitors. Strong spatial heterogeneity can preserve diversity by diminishing the chance of fixation for advantageous mutations; however, this barrier can be overcome if the mutant has improved migration capability (Manem et al. 2015).

Niche partitioning can also be enabled by different ecological strategies of coexisting groups. For example, coexistence between different marine bacterioplankton groups of *V. cyclitrophicus* in a patchy nutrient environment may be supported by different utilisation/dispersal strategies: one group specialising in accessing localised resources within each patch and the other group specialising in dispersing and discovering new patches (Yawata et al. 2014).

### 4.2 Successional Niche Occupation

Niche partitioning can also happen through temporal succession. For example, spatial dynamics can contribute to coexistence by allowing successive colonisation of patches by different groups, a process called successional niche occupation. Conceptually, this can be considered to be another manifestation of niche partitioning—in time rather than space—enabled by dispersal among interconnected patches. Hassell et al., for example, showed that dispersal in a patchy environment can support the coexistence of competitors, even if patches are identical. They interpreted this phenomenon as self‐organised spatial dynamics (Hassell et al. 1994). Similarly, spatial heterogeneity has been shown to allow the stabilisation of predator–prey interactions by allowing spatial dynamics across interconnected patches (Petrenko et al. 2020). Successional niche occupation can also involve the generation of new spatial niches by other groups. For example, early colonisers in oral microbiota generate a new niche by creating opportunities for cell adhesion for the successors (Kuramitsu et al. 2007; Jakubovics and Kolenbrander 2010).

### 4.3 Loss of Coexistence due to Spatial Isolation

A pronounced spatial structure may not always support more coexistence. For example, slower diffusion of interaction mediators in the environment—making the effect of the spatial structure stronger—also weakens inter‐group facilitation and may lead to less coexistence overall (Lobanov et al. 2023; Saxer et al. 2009). Snyder and Chesson (2004) examined the interplay between dispersal, competition and environmental heterogeneity and found that, depending on the scales of different processes, the overall effect could either promote or suppress coexistence.

### 4.4 Group‐Driven Spatial Self‐Organisation

In addition to spatial heterogeneity of the environment itself, spatial self‐organisation emerging from species activities can also generate or modify niches and influence coexistence. For instance, in a community of antibiotic‐producers, antibiotic‐sensitive and antibiotic‐resistant bacteria, spatial patterns emerged, driven by interactions through antibiotics, that allowed coexistence (Narisawa et al. 2008).

**FIGURE 4:**
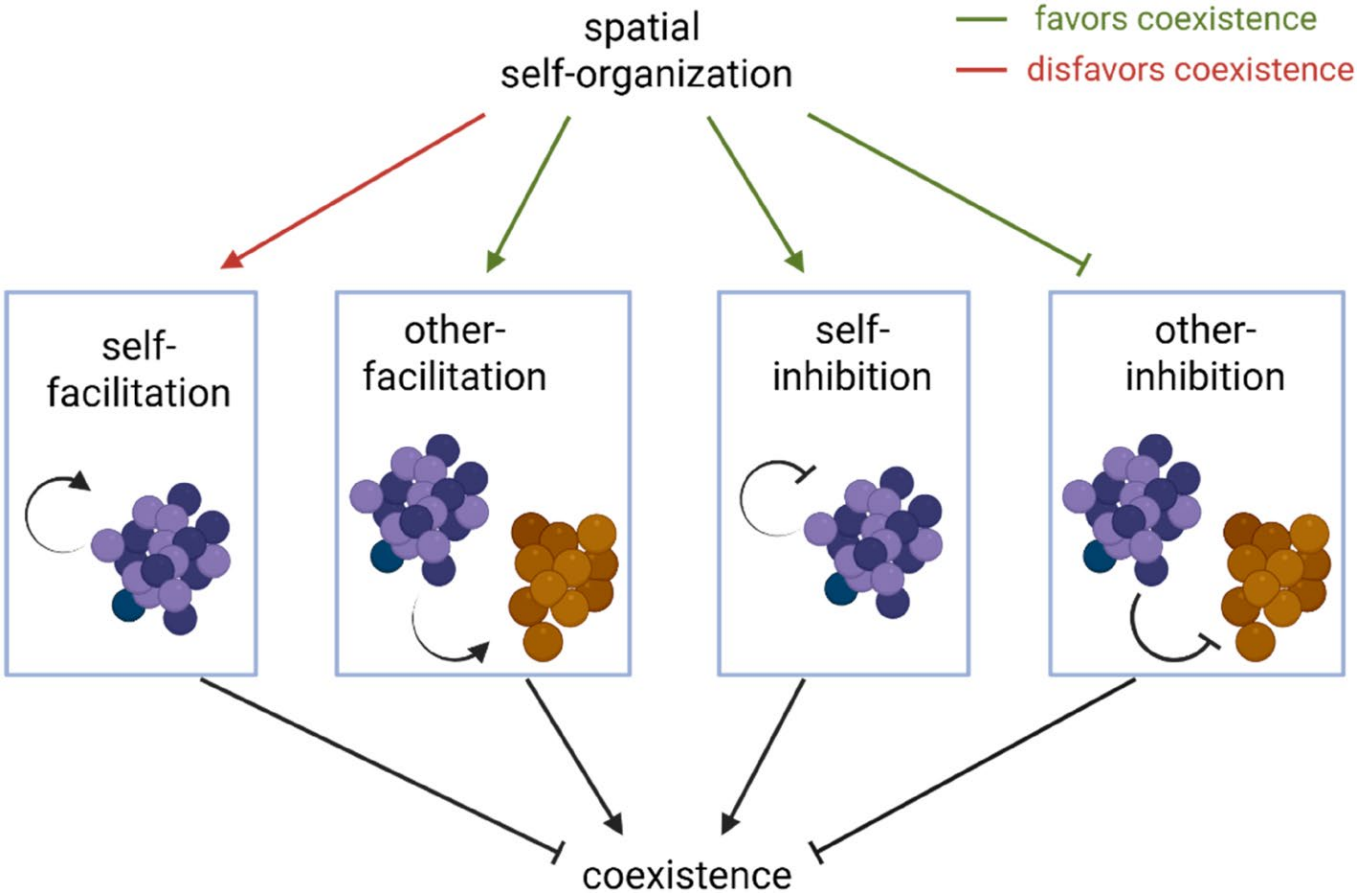
Spatial self‐organisation can impact coexistence by changing the effective strength of interactions. Spatial self‐organisation is expected to strengthen facilitation (both for self and others) and self‐inhibition, but to weaken the inhibition of other groups. Given that self‐facilitation and other‐inhibition are expected to disrupt coexistence, overall, spatial self‐organisation is expected to disfavour coexistence by supporting self‐facilitation and favour coexistence by supporting other types of interactions.

In a simplified view, three prominent trends can contribute to higher coexistence and diversity in spatial communities (Figure 4): (1) magnifying intra‐group competition for resources over inter‐group competition (Stoll and Prati 2001), (2) reinforcing inter‐group facilitation by spatial intermixing (Momeni, Brileya, et al. 2013), and (3) suppressing inter‐group inhibitory effects by spatial segregation of antagonistic groups (Kreth et al. 2005). Coexistence can also be driven by spatial patterns arising from interactions between the groups and their environment. For example, in biofilms of *Klebsiella pneumoniae* and *Pseudomonas aeruginosa* , different attachment and detachment rates can balance the competitive advantage and allow coexistence (Stewart et al. 1997).

Emergent spatial patterns can also act against coexistence. For example, in a community of cooperators and cheaters, self‐organisation and intermixing of cooperators can spatially isolate cheaters and drive them to extinction (Momeni, Waite, and Shou 2013). Spatial structure can also amplify self‐facilitation—a group converting resources to a beneficial factor for its own—which can be useful for maintaining competitively disadvantaged groups but can also act as a destabilising force by providing positive feedback (Lobanov et al. 2023).

## 5 The Impact of Spatial Structure Through the Lens of Mathematical Models

### 5.1 Overview of Modelling Approaches

Mathematical modelling offers a complementary source of insights, enabling more control to set up desired assumptions and arrangements and more transparency to facilitate the interpretation of the outcomes. These, in turn, allow more mechanistic investigations into how and by how much the spatial context contributes to community outcomes. Below, we briefly summarise a few conceptual models, each capable of capturing the relevant biological processes and the impact of spatial structure within a range of scales and assumptions. Even though we present these models as separate entities, a mathematical model can be a combination of these models, capturing each relevant process using a corresponding model at an appropriate scale and level of abstraction.

There are different levels of simplification to capture the main aspects of spatial structure, and a choice can be made on how to represent the system. As Durrett and Levin (1994) put it, “[this choice] goes far beyond the mathematical convenience to the heart of understanding the mechanism,” especially to unravel what mechanisms are important for explaining spatial phenomena of interest.

At one extreme, the simplest representation is a mean‐field model, which captures how, on average, the spatial structure affects the groups experiencing that environment. When a global average adequately represents the net effect on a group in a spatial context, the mean‐field model offers significant simplification. However, the expectation is that when there is significant heterogeneity in the environment, significant structure in group distributions, or short‐range interactions are dominant, a mean‐field model may not capture the details of spatial distribution.

Patch models, predominantly used in ecological studies, refer to representations in which the environment consists of well‐mixed patches connected by a network of links between patches. With limited dispersal, the environment will become structured as individuals within the same patch interact more strongly with each other compared to individuals residing in different patches (Pacala et al. 1990).

Recognising diffusion as a primary process for microbial dispersal and chemical spread in the environment, reaction–diffusion models are often utilised (Momeni, Brileya, et al. 2013; Lobanov et al. 2023). The essence of these models considers agents that can move or spread in the environment (e.g., via random walk (Berg 1993)) and interact with other agents through defined reactions. These models can also capture how chemical compounds, such as nutrients, toxins and metabolic byproducts, mediate microbial interactions (Dukovski et al. 2021; Niehaus et al. 2019).

A more explicit approach is to explicitly simulate individual cells/units within a group, along with the relevant processes for their interactions in an agent‐based model (Dang et al. 2020; Maire and Youk 2015; Kreft et al. 2001; Grimm and Railsback 2005; Ferrer et al. 2008). Keeping track of individuals, their unique environment and possibly even their unique properties (or behaviours) allows a detailed representation of the microbes in a spatial context. Such a detailed presentation of patterns and dynamics can reveal complex dynamics emerging from individual‐level rules. The downside is that such models require an understanding of the detailed mechanistic rules that apply to individuals and are often computationally intensive.

### 5.2 Computational Platforms and In Silico Experiments

Several open‐source software implementations use agent‐based and individual‐based representations of spatial microbial systems and provide practical tools for exploring the concepts discussed above. For example, COMETS couples genome‐scale metabolic models with spatial diffusion and growth and has been widely used to study cross‐feeding, metabolic competition and spatially mediated coexistence (Dukovski et al. 2021). CellModeller focuses on explicit single‐cell growth, division and mechanics, making it particularly useful for investigating how cell‐level rules give rise to collective spatial patterns (Rudge et al. 2012). iDynoMiCS emphasises biofilm formation and chemical microenvironments and has been extensively applied to questions of spatial heterogeneity, resource gradients and multispecies structure (Lardon et al. 2011).

Beyond serving as simulation frameworks, these tools enable in silico experiments in which mechanistic assumptions can be systematically perturbed while holding others fixed. Such computational experiments have been used, for example, to test how interaction ranges, diffusion rates, or spatial arrangement affect coexistence, cooperation and pattern formation, complementing laboratory experiments where these variables are often difficult to control independently. While these platforms differ in scope and biological detail, they share a common role in translating conceptual models of spatial interactions into testable, mechanistically explicit predictions, thereby linking theory, computation and experiment.

## 6 Outlook: What Is Next?

### 6.1 Measuring Spatial Patterns in Nature

Although the impact of spatial structure in microbial communities has been studied in many examples, there is still room to explore the mechanisms involved in the emergence and maintenance of community patterns. A necessary step for proper interpretation of the role of space is developing the tools and techniques to observe the spatial details of communities. Recent advances in multimodal and multiplexed imaging are one of the significant steps forward, allowing detailed characterisation of the spatial positioning of cells (Valm et al. 2011; Welch et al. 2016) and the spatial profile of the chemical environment (Watrous et al. 2013; Alcolombri et al. 2022). Advances in sequencing have also complemented this progress by offering the capability to monitor the spatial distribution of microbial populations (Sheth et al. 2019), proteins and metabolites.

### 6.2 Controlling Spatial Structure and Pattern

A major shift in recent years has been developing to make laboratory‐based studies more realistic. An important aspect of such efforts involves better representation of the intrinsic spatial structure of the corresponding biological system. A variety of examples, from artificial soil to organ‐on‐a‐chip devices, underscore the recent advances and highlight the impact of the spatial context (Del Valle et al. 2022; Mafla‐Endara et al. 2021; Thacker et al. 2020).

The technical difficulties of controlling the spatial arrangement of cells pose limitations on how detailed studies can be designed for spatial studies. Advances in microfluidics (Kim et al. 2008; Kim et al. 2011) and bioprinting (Lan et al. 2018; Mohammadi and Rabbani 2018) have already taken steps to address this shortcoming. Recent technological improvements in these areas have made these strategies more accessible and broadly applicable.

### 6.3 Designing Community Functions in a Spatial Context

An important step towards implementing synthetic communities with desired functions is to utilise the spatial context for stability or enhanced functionality. For example, Kim et al. (2011) implemented pre‐structured synthetic communities to control community processes and achieve efficient degradation of organic pollutants in the presence of inhibitory heavy metals. Beyond pre‐structuring, programmed pattern formation can be viewed as an additional design opportunity (Lu et al. 2022). Maintaining microbial populations is thought to be a main challenge in consolidating bioprocessing (Olson et al. 2012), and programmed pattern formation can offer a solution for maintaining important community functions.

### 6.4 Accounting for Evolution in a Spatial Context

A better understanding of coexistence in a spatially structured environment cannot be achieved without considering evolutionary processes (Yanni et al. 2019). There are several ways that spatial structure can impact the trajectory of evolution and coevolution. Spatial structure can create a niche for the rise of certain mutations and genotypes (Nadell et al. 2016). For example, Chao and Levin (1981) showed that a toxin‐producer genotype was disfavoured in well‐mixed environments and could arise only when the spatial structure localised the competitive benefits to the toxin‐producing genotype. In phage‐bacteria dynamics, Kerr et al. (2006) showed that unrestricted dispersal selected for more aggressive phage and loss of productivity, whereas restricted migration favoured prudent phage and averted the tragedy of the commons. Spatial structure can also reshape inter‐group interactions. For example, Hansen et al. (2007) demonstrated that the interaction between *Pseudomonas* and *Acinetobacter* in a community transitioned from commensalism to exploitation only in a spatially structured environment. The evolution of spatially relevant traits is another aspect enabled by the spatial context. For instance, Parvinen et al. (2023) have shown that under different circumstances, such as the presence or absence of temporal heterogeneity, spatial heterogeneity can have opposing effects on the evolution of dispersal. Lastly, as an example of coevolution, Hochberg and van Baalen have examined the evolution of a prey–predator system in a patchy system and found that within‐group diversity was highest in habitats at intermediate levels of productivity (Hochberg and Van Baalen 1998). Although not comprehensive, the above examples serve as snapshots of some of the ways through which evolutionary processes both impact the spatial structure and are impacted by it.

### 6.5 Concluding Remarks

Studying microbial communities is motivated by the need to understand the mechanisms involved in microbiota functions and ecosystem processes. Given that the spatial context is often an intrinsic feature of microbial communities, here we have highlighted what processes the spatial structure stems from, how the spatial context affects interactions among different groups within a community and how the spatial structure influences community properties, including properties as fundamental as the coexistence of community members. In our treatment of the spatial structure and its role, we have used conceptual simplifications to find common threads among diverse and often complex microbial systems. Rather than delving into the complexities of each system, we simplified the representation by focusing on general concepts such as the localisation of groups, average distance among interacting groups and interaction‐driven self‐organisation. These simplifications, in our opinion, provide intuitive—even if not fully accurate—guidelines about the essence, emergence and progression of spatial structure for microbial communities. The next milestone, in our opinion, is to take this insight and apply it to control the structure and function of microbial communities. Towards this goal, recent progress in quantifying the extent and impact of spatial structure, in controlling the distribution of microbial groups within a community, and in incorporating microbial ecology and evolution in designing spatial communities are important and promising steps.

## Author Contributions

**Vaishnavi Warrier:** writing – review and editing, writing – original draft, investigation, conceptualiation. **Yilin Chen:** investigation, writing – original draft. **Ethan Rappaport:** investigation, writing – original draft, writing – review and editing. **Shin Haruta:** conceptualization, writing – review and editing. **Hyun Youk:** conceptualization, writing – original draft, writing – review and editing, supervision. **Babak Momeni:** conceptualization, investigation, funding acquisition, writing – original draft, writing – review and editing, visualization.

## Funding

This work was supported by the National Science Foundation, 2430384; Boston College, Undergraduate Research Fellowship; and National Institutes of Health, NIH‐NIGMS R35, GM147508.

## Conflicts of Interest

The authors declare no conflicts of interest.

## Data Availability

Data sharing not applicable to this article as no datasets were generated or analysed during the current study.
